# Farmers’ response to COVID-19 disruptions: The case of cropland allocation decision

**DOI:** 10.1016/j.sftr.2022.100088

**Published:** 2022

**Authors:** Edward Martey, Peter Goldsmith, Prince M. Etwire

**Affiliations:** a Socio-economic Section, CSIR-Savanna Agricultural Research Institute, P.O. Box TL 52, Tamale, Ghana; b University of Illinois at Urbana-Champaign, 414 Mumford Hall, 1301 W Gregory Drive, Urbana, IL, USA

**Keywords:** COVID-19 pandemic, Crop land allocation, Commercial and staple crops, Seemingly unrelated regression

## Abstract

This study provides new evidence on the determining factors of cropland allocation decisions within the context of the COVID-19 pandemic by using the seemingly unrelated regression (SUR) model on 309 farm households. The results reveal that socio-economic, production, institutional, and political factors significantly influence the choice and size of cropland allocation decisions among legumes and cereals. Beyond these factors, we find that COVID-19 education increases land area allocated to staples while perception of disruptive effect of COVID-19 on agriculture positively correlates with the area under commercial crop (soybean) production. The implications of the results are discussed.

## Highlights

• The study investigates farmers’ response to the COVID-19 pandemic in terms of land allocation decisions.
• The study uses farmer survey in Ghana.
• Farmers exposed to COVID-19 education allocate more land to production of staples (maize and rice).
• Farmers who anticipate a disruptive effect of COVID-19 put more land under commercial crop production (soybean and groundnut).
• COVID-19 education and understanding of farmers’ perceptions are important to guide future adaptation and mitigation strategies.

## 1 Introduction

The novel COVID-19 pandemic is a global emergency affecting all countries with older people, poor households, the undernourished, and those who live in remote areas being the most affected [1]. This pandemic has resulted in a huge economic shock leading to disruption in the agricultural sector and the food supply chains. The pandemic has resulted in several deaths leading to loss of human capital and labor (Abate et al., 2020; [2]). In response to the COVID-19 pandemic, the World Health Organization (WHO) encouraged member countries to implement strategies such as lockdown, social distancing, washing of hands under running water, etc. The lockdown strategy had both positive and negative consequences on the livelihoods of people.

The agricultural production, distribution, and marketing sectors were expected to be disrupted [1,2]. For, example the non-availability of migrant labor will interrupt planting and harvesting activities in the immediate future. Production of food crops is expected to be affected negatively, therefore limiting the food systems with devastating impacts on food security [2]. The reduction in food supply to the urban centers may result in price hikes. Transportation of food to the urban centers is affected due to the disruptions in the transportation sector and large agribusiness input firms may partially withdraw leading to high input prices [1]. The partial lockdown imposed in some EU, the United States, Asia, and African countries are likely to impact negatively on the transfer of remittances to rural households.

In Ghana, the COVID-19 pandemic affected the two most commercial regions – Greater Accra and Ashanti regions. Most of the food production occurs in northern Ghana while the southern part of Ghana (Greater Accra and Ashanti regions) is recognized as the consumption centers. A previous study by Martey et al. (2019) indicates that markets in Ashanti and Northern regions serve as lead production and demand markets, respectively. Any trade disruptions (for example, reduction in the supply of agricultural inputs and volume of trade) between these markets due to external shock are likely to disrupt the agricultural production systems. A published report by the Ghana Statistical Service (GSS) indicates that inflation rose from 10.6% in April to 11.3% in May, 2020. These price hikes have been attributed to the partial lock-down which spanned for three weeks. However, an increase in food prices have contributed largely to the current inflation figures. Based on regional disaggregation, Greater Accra recorded the highest inflation (13.3%) relative to the other regions (3.1−9%). Food inflation contributes close to 60% of the May inflation figure (Ghana Statistical Service^1^, 2020).

The rural agricultural sector may be affected to the extent that farmers may strategize by reallocating land resource between staples (cereals - maize and rice) and commercial crops (legumes - soybean and groundnut) in response to high food prices. Several studies have shown that smallholder farmers are more likely to use strategies that allow them to mitigate exogenous shocks ([3]; Martey et al. 2020; Tambo 2016). For example, [3] find that farmers in Vhembe District of South Africa respond to climate shock by adopting drought-tolerant seeds, shorter cycle crops, diversification of crops, changing planting dates, small-scale irrigation, migrating to urban areas, and involvement in petty trading. A study by show that farmers’ decision to shift to towards market-oriented production systems is strongly influenced by changes in the norms of disease management, influential factors (government technicians/head of the village), and the taste and preferences of urban inhabitants. Martey et al. (2020) find that perceptions of climate variability and shocks have a heterogeneous effect on the adoption of multiple integrated soil fertility management practices (ISFM) practices. [4] find that farmers in developing countries diversify their crop portfolios as a mitigation strategy against climate variability but [5,^6^,7], indicate that the intensity of crop diversification differ from farmer to farmer due to the differences in the production capacity and perception of climate variability.

While food price hike poses a serious threat to household food security, farmers are likely to benefit from the situation by becoming more market oriented. Farmers may respond to the price hike by reallocating land resources between commercial and staple crops. Therefore, our study hypothesizes that farmers respond positively to the price hike due to the COVID-19 pandemic by increasing area under commercial crops. We empirically test this hypothesis by using the seemingly unrelated regression (SUR) model to identify the factors influencing crop land allocation decision between cereals and legumes. Several studies [8,^9^,^10^,11] have highlighted the factors influencing cropland allocation decisions in Asia and SSA with limited evidence on how external health shocks influence cropland allocation decisions for cereals and legumes within a developing context. The novel COVID-19 pandemic presents a unique case to ascertain the correlation of health shocks on cropland allocation decisions.

This study makes two main contributions to agricultural land use resource literature. First, it explores the level of farmers’ awareness regarding the disruptive effect of COVID-19 on agricultural production. We find that a relatively high number of farmers are aware of the COVID-19 pandemic but have limited information on the effect on agriculture as well as the preventive measures to contain the spread. Second, the paper extends the literature by assessing the correlation between COVID-19 and cropland use for staple and commercial crops. The results indicate that COVID-19 education is positively correlated with land area allocated to staple production relative to commercial crop production while farmers’ perception that COVID-19 will impact negatively (disruptions in the factor input market, food system, and transportation sector) on agricultural production leads to an increase in area under commercial crop production.

The rest of the paper is structured as follows: section 2 presents the rationale for legume and cereal production followed by the conceptual and theoretical framework and empirical strategy in section 3; in section 4, the sampling, data and summary statistics are presented while section 5 discusses the empirical results; concluding remarks and policy recommendations are provided in section 6.

## 2 Rationale for commercial and staple crop production within a developing context

Production of commercial (such as legumes) and staple (such as cereals) crops by smallholder farmers are generally motivated by several factors. The primary rationale regarding smallholder farmers’ intercropping behavior of cereals with legumes is for soil conservation and improvement of soil fertility, profit maximization, risk minimization, and weeds and pest control. This is important especially within a developing context like sub-Saharan Africa (SSA) where there are erratic rainfall and low input use.

Farm households cultivate staples (cereals) for storage or consumed on-farm or sold to various market outlets. Staple foods require better coordination strategies between farmers and agro-processors to enhance the shelf-life. However, investment in staple food is constrained by the perception of low investment and higher risks for farmers and private agribusiness firms [12]. The challenge with staple crops enterprise is that they consist of a large and highly heterogeneous number of small-scale producers with women as the important players in production, trading, and small-scale agro-processing [12].

Commercial crops such as legumes are mainly cultivated as a cash crop. Farmers perceive the cultivation of commercial crops as a way of integrating into the market to hedge against external and uncontrollable changes [13]. For example, legumes are a major source of feed for livestock and a valuable complement to primarily carbohydrate-based diet for humans given that they have high protein and micronutrients [14,^15^,16]. Legumes provide a range of biophysical benefits such as yield improvement through biological nitrogen fixation (BNF), soil fertility enhancement, and erosion control [17,^18^,^19^,^20^,21]. The crop can generally be stored for a long period of time relative to root and tuber crops which are highly perishable. The low perishability of legumes enhances its commercialization between harvest thus reducing the risk of households becoming cash trapped [22,23]. Soybean is relatively new and associated with high fixed-costs related to learning new production practices and input utilization methods, and high capital investments in mechanization and market linkages [24]. Similarly, smaller farm households may not find soybean profitable if they are unable to spread their fixed costs over larger areas of land and attract buyers who will provide a competitive price for the grain [25].

Some studies have shown that the cultivation and use of legumes in smallholder farms is influenced by socio-economic factors such as gender, income, limited and uncertain market and transportation access, limited access to improved varieties, lack of reliable supply of quality seed, and farmers’ perceptions of legume attributes [13,^26^,^27^,^28^,29]. Martey et al. (2020) find that soybean production in Northern Ghana is a response to high demand in the consumption areas of Ghana. In Kenya, [30] show that some farmers buy staple food crops from income generated through the cultivation and sale of cash crops. Farmers respond to higher domestic food prices by shifting land allocation decisions between crops (Eldukherv et al., 2010).

## 3 Conceptual framework and empirical strategy

### 3.1 Conceptual framework

**Fig. 1:**
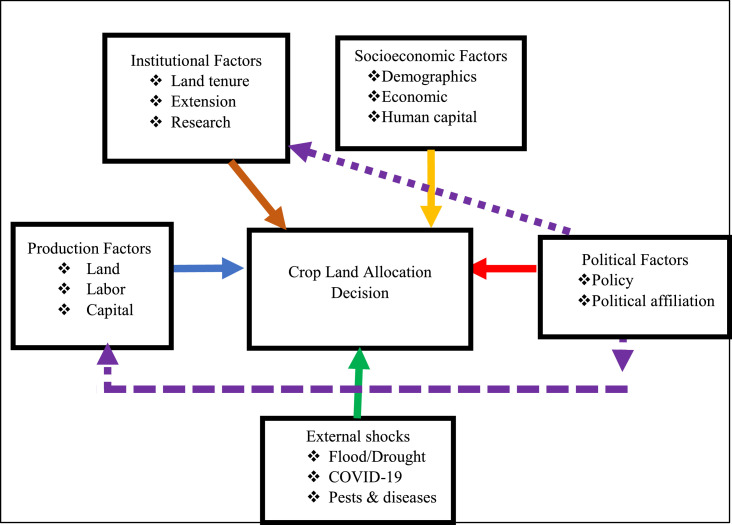
Conceptual framework (Authors’ construct).

Fig. 1 shows the conceptual framework of the study. Agricultural land allocation is a rational decision-making process among smallholder farmers which has long-term effect on agricultural development and poverty reduction [31]. Farm households derive their main source of livelihood from agricultural production with land playing a critical role in the production process. A household may allocate land among different crops with the expectation of achieving food security given that land is fixed. However, the objective of the farm household determines the extent to which agricultural land is distributed among crops. A farm household whose objective is food security will allocate more land to food crops such as cereals (maize and rice) while those whose objective is income or profit maximization are likely to allocate more land to commercial crops such as legumes (soybean and groundnut). Nonetheless, some households may allocate land among different crops as a risk-reducing strategy with the ultimate objective of enhancing the welfare outcome of household members [32].

Understanding land allocation decisions are critical for formulating evidenced-based agricultural policies since land is a fundamental input for production, especially among smallholder farmers. This study hypothesizes that land allocation decision is influenced by variables such as institutional, political, socio-economic, and production factors and external shocks [8]. Production factors include land, management, capital, and labor. These factors are assumed to be used in equal proportion across the same crop category. Farmers will allocate more land to the crop category that maximizes either profit or utility. Political factors include policies, political capital, and relationship with political figures. For example, policies that favour cereal production will witness a higher response by farm households by allocating more land to cereal production. Similarly, farmers who are related to political figures are more likely to benefit from agricultural development projects that promote specific crops. Socio-economic factors such as sex, education, age, total dependents, etc. are likely to influence land allocation among different crop types.^2^

Institutional factors such as land tenure system and access to extension and research have a direct effect on food security and livelihood activities and strategies through land resource allocation [33]. External shocks such as climate, pests and diseases, and pandemics such as the novel Coronavirus may influence land allocation decision in several ways. Farmers are more likely to allocate more land to legumes when the expected future price is relatively higher than cereals. Partial lockdowns and restrictions in the movement of goods and services disrupt food and input distribution systems. Disruption of trading during the lockdowns and other restrictions can lead to a decline in the market price of food commodities thus influencing land allocation decisions of the affected crop types. We hypothesize that farmers will respond more favorably towards a crop category if the COVID-19 pandemic leads to market opportunities such as increase in output price, decrease in input price, and high demand.

### 3.2 Empirical model

Farm households allocate their available cropland to four major crops – maize, soybean, rice, and maize, therefore the probability of allocating a specific field to a crop may be correlated with the land allocated to other crops. The decision to allocate land to a specific crop is determined by exogenous factors that might be the same or differ among crops. Modeling of land allocation decision requires a model that accounts for the correlation of the error terms among the set of equations. The crops were grouped into two categories: (1) cereals (maize and rice) and (2) legumes (soybean and groundnut) given that not all households grow the same crop types. The study assumes that technology, inputs, output prices, and time allocation are homogeneous with crop categories.

Land allocation decision is expected to be influenced by factors such as socio-economic (X), production (R), political (M), institutional (I), and external shock (E) (Fig. 1). Following the specification of Zellner (1962) and [8], the empirical model is specified as follows:

(1) $$\left\{ \begin{array}{c} y_{i1}=\gamma_{1}X_{i1}+\beta_{1}R_{i1}+\alpha_{1}M_{i1}+\theta_{1}I_{i1}+\delta_{1}E_{i1}+e_{i1}\\ \;\\ y_{i2}=\gamma_{2}X_{i2}+\beta_{2}R_{i2}+\alpha_{2}M_{i2}+\theta_{2}I_{i2}+\delta_{2}E_{i2}+e_{i2} \end{array}\right.$$

where $\gamma_{m}$, $\beta_{m}$, $\alpha_{m}$, $\theta_{m}$, and $\delta_{m}$ are the parameters to be estimated; $e_{im}$ are the error terms. The parameters are estimated using the Ordinary Least Square (OLS) method through a “Seemingly Unrelated Regression” (SUR).

## 4 Sampling, data and summary statistics

### 4.1 Sampling and data

**Fig. 2:**
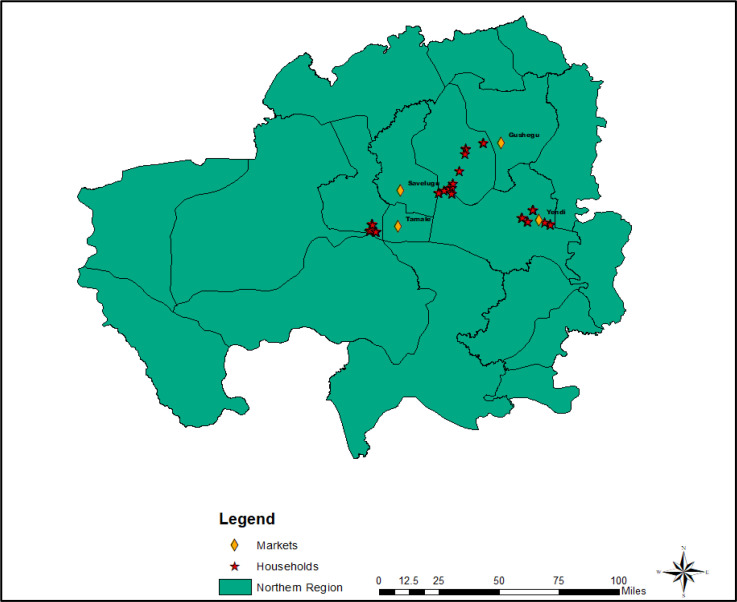
Administrative map of former Northern Region of Ghana.

Fig. 2 shows the Northern Region of Ghana where the study was carried out. The region used to have 26 districts with a land size of 70,384 square kilometers. In 2019, two new administrative regions (i.e., North East and Savannah) were carved out of the Northern Region bringing the total number to three. Generally, the climate of the region is relatively dry, with a single rainy season that begins in May and ends in October with an average annual rainfall of 750 to 1050 mm, while the dry season, with temperatures of up to 38°, occurs between November and April. The dominant crops cultivated include maize, yam, millet, sorghum, rice, groundnuts, soybean, and cowpea.

The survey was carried out in four districts namely Tolon, Gushegu, Karaga, and Yendi. A multi-stage sampling technique was used in the sampling of the farmers. The first stage involves a purposive selection of four districts in the Northern Region due to the high number of cereal and legume farmers in the selected region and districts. This was followed by a purposive sampling of 15 communities distributed across the four selected districts. The purposive selection of the communities was based on the size of the district, the population of cereal and legume farmers and the level of crop diversification. Finally, a total of 309 farmers were sampled from the 15 communities using a simple random sampling technique.

Our analysis of land allocation decision is based on the June 2020 planting season. The data collected captured household demographic and farm characteristics, fertilizer use, access to social amenities, crop production, sustainable agricultural practices, distribution of farm output, improved seed variety adoption, constraints to crop production, and risk reducing strategies such as the purchase of insurance policy against crop failure. The data captured COVID-19 related information such as level of awareness, education on COVID-19, perceptions of the effect of COVID-19 on agricultural production, food security, and land allocation decisions.

### 4.2 Summary statistics

Table 1 shows the summary characteristics of the explanatory variables used in the model. The results of the study show that 78% of the sampled farmers are males and the mean age and years of schooling are 41 years and two years, repectively. The distribution of the household members shows an average of two household members across all the different age categories. A sampled farmer has eight dependents on the average and 93% of the sampled farmers are natives which guarantees access to communal resources such as land for agricultural production. About 27% of the sampled farmers belong to a farmer-based organization while 20% hold a leadership position in such association. With respect to institutional variables, the data show that 28% and 77% of the sampled farmers are related to a political figure and affiliated with a political party which enhances their chance of being considered for developmental projects in the communities. About 50% of the sampled farmers engage in other economic activities apart from farming. About 37%, 20%, 19%, and 11% of the sampled farmers have access to input shop, grain market, credit, and research institute. Farmers in our sample have 18 years of farming experience. While 32% of the respondents have access to extension services, 2% and 10% have insured their farm and engage in contract farming.

**Table 1:** Summary statistics of respondents.

Variable	Mean	Std. Dev.	Min	Max
Sex (1=male)	0.783	0.413	0	1
Age	40.618	12.215	20	85
Years of education	2.181	4.150	0	16
Number of males less than 5 years	1.942	1.757	0	12
Number of females less than 5 years	1.706	1.514	0	10
Number of males between 6 and 14 years	1.926	1.735	0	12
Number of females between 6 and 14 years	1.680	1.509	0	8
Number of males between 15 and 35yrs	2.110	1.836	0	15
Number of females between 15 and 35yrs	2.304	1.872	0	13
Number of males between 36 and 60 years	1.285	1.061	0	5
Number of females between 36 and 60 years	1.437	1.282	0	8
Number of males above 60 years	0.333	0.537	0	2
Number of females above 60 years	0.256	0.589	0	4
Total dependents	8.443	6.783	0	39
Nativity (1=native)	0.926	0.263	0	1
Member of FBO (1=yes)	0.265	0.442	0	1
Hold leadership position (1=yes)	0.197	0.399	0	1
Related to political figure (1=yes)	0.275	0.447	0	1
Affiliated to political party (1=yes)	0.767	0.423	0	1
Engaged in other economic activity (1=yes)	0.498	0.501	0	1
Access to input shop (1=yes)	0.369	0.483	0	1
Access to grain market (1=yes)	0.204	0.404	0	1
Access to credit	0.194	0.396	0	1
Access to research institute (1=yes)	0.110	0.313	0	1
Years of farming	18.104	10.546	2	48
Access to extension services (1=yes)	0.320	0.467	0	1
Farm insured (1=yes)	0.019	0.138	0	1
Contract farming (1=yes)	0.104	0.305	0	1

Table 2 shows the gender disaggregated effect of COVID-19 on agricultural production. Males are generally involved in the cultivation of more crops and as a strategy to cope with COVID-19, males increase their farm size to a variety of crops. Based on the result, most males (44.68%) increased the farm size allocated to the cultivation of soybean. About 28% and 2% of males allocated more land to groundnut and rice, respectively due to COVID-19. On the contrary, all the sampled female farmers allocate more land to soybean cultivation. The perceived effect of COVID-19 on agricultural production by farmers was mixed. A large proportion (68%) of male farmers perceived COVID-19 to have a negative (disruptions in the factor input market, food system and transportation sector) effect on agricultural production. About 26% of male farmers perceive COVID-19 to have both negative and positive effects on agricultural production. Alternatively, 48% of female farmers perceived COVID-19 to have both positive and negative effects on agricultural production while 39% indicated that the effect of COVID-19 on agricultural production is negative. The chi-square test shows that there is a significant association between the sex of a farmer and their perceived effect of COVID-19 on agricultural production. The main sources of sensitization on COVID-19 for both male and female farmers are non-governmental organizations (NGOs) and agricultural extension agents (AEAs). Most farmers (50% males and 58% females) reported soybean as the most profitable crop due to COVID-19 due to inflationary price effects in the consumption areas of Ghana.

**Table 2:** COVID-19 effect on agricultural production.

Variable	Male	Female	Chi2
*Increase crop farm size due to COVID-19*			
Maize	25.33	0.00	2.36
Soybean	44.68	100.00	
Rice	2.13	0.00	
Groundnut	27.66	0.00	
*Effect of COVID-19 on agricultural production*			
Negatively	67.77	38.81	18.63***
Positively	6.61	13.43	
Both	25.62	47.76	
*Source of sensitization of COVID-19*			
AEAs	41.10	41.67	2.18
NGOs	50.68	58.33	
Research Organization	8.22	0.00	
*Most profitable crop due to COVID-19*			
Soybean	50.41	58.21	4.29
Maize	28.51	16.42	
Groundnut	19.42	22.39	
Rice	1.65	2.99	

## 5 Results and discussion

### 5.1 Farmer awareness and perceptions of COVID-19 pandemic on farm production

Table 3 reports farmers’ awareness of COVID-19 and their perceptions of COVI-19 related factors on agricultural production. About 98% of farmers are aware of COVID-19. Given the high level of awareness, it is expected that farmers may take safety precautionary steps to curb the spread of the virus and strategically plan their farming activities. A similar result was also observed for both men (98.3%) and women farmers (95.5%). Despite the high level of awareness, the data show that only 33% of the farmers have been educated or sensitized on the COVID-19 safety protocols. These sensitizations were done by AEAs, NGOs, and research organizations. The result indicates the need for further education to reduce the potential risk of community spread and impact of the COVID-19 on agricultural production.

**Table 3:** Awareness and perceptions of COVI-19 pandemic on crop production.

	Males		Females		Pooled	
Variable	Mean	Std. Dev.	Mean	Std. Dev.	Mean	Std. Dev.
Awareness of COVID-19	0.983	0.128	0.955	0.208	0.977	0.149
COVID-19 will influence agriculture	0.640	0.481	0.373	0.487	0.583	0.494
Sensitization/education on COVID-19	0.331	0.471	0.358	0.483	0.337	0.473
COVID-19 influences crop production decision	0.116	0.321	0.104	0.308	0.113	0.317
Current market price influence production decisions	0.157	0.365	0.134	0.344	0.152	0.360
Allocation of more land to a specific crop	0.240	0.428	0.134	0.344	0.217	0.413
Current crop cultivated due to COVID-19	0.050	0.218	0.104	0.308	0.061	0.241
Readily available labor	0.595	0.492	0.388	0.491	0.550	0.498
More family labor use than previous season	0.450	0.499	0.493	0.504	0.460	0.499
Observation	242		67		309	

About 58% of the respondents opined that COVID-19 will influence agricultural production and 11.3% of the farmers indicate that COVID-19 influences their current production decisions. The farmers anticipate that lack of buyers, high cost of laborers, and high input prices will affect the current production decisions. The result shows that about 36% of the farmers indicated that their production decisions for the 2020 cropping season were influenced by the current market prices of food and commercial crops. This indicates that farmers were responding positively by cultivating crops with relatively high output prices. Generally, relatively few farmers (41.3%) allocated more land for the cultivation of soybean and maize. Comparatively, the allocation of more land for the cultivation of specific crops was higher among men (24.0%) than women (13.4%). Less than 7% of the farmers changed their cropping pattern due to COVID-19 and 55% stated that labor for agricultural production is readily available. About 46% of the sampled farmers indicate that more family labor was used in the previous cropping season (2019) relative to the current cropping season (2020). The use of more family labor for agricultural production in the previous cropping season is relatively higher among women (49%) than men (45%).

**Fig. 3:**
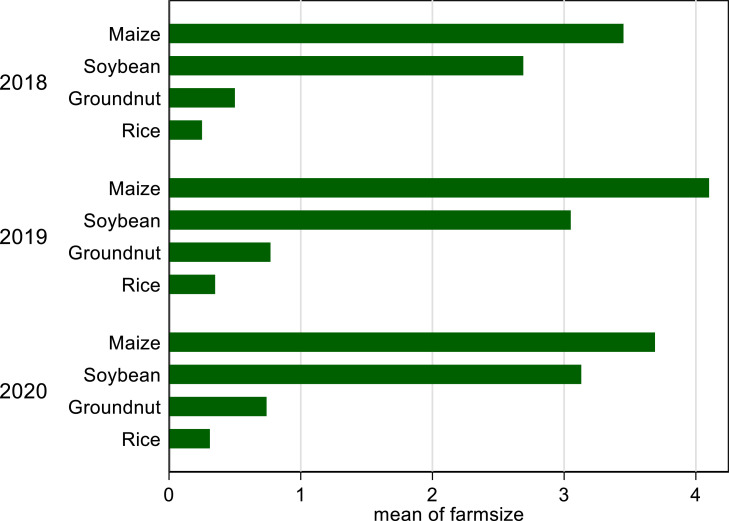
Land allocation decisions across crop types and year.

Fig. 3 shows the change in land allocation decisions for maize, soybean, groundnut, and rice from 2018 to 2020 cropping season. Generally, the area under maize cultivation is relatively higher than the area under soybean production. The area allocated to soybean production in 2018, 2019, and 2020 are 2.69 acres, 3.05 acres, and 3.13, respectively. Similarly, the area allocated to maize production increased from 3.45 acres in 2018 to 4.1 acres in 2019 but decreased to 3.69 acres in 2020. The relatively high acreage of maize highlights the importance of maize to farmers in the study area. However, in 2020 the result shows a change in the land allocation decision between maize and soybean. While the area allocated to maize production declined, the area under soybean cultivation increased.

The area allocated to groundnut and rice cultivation from 2018 to 2020 is less than one acre. The area under groundnut production increased from 0.50 acres in 2018 to 0.77 acres in 2019 and decreased to 0.74 acres in 2020. The area under rice cultivation increased from 0.25 acres in 2018 to 0.35 acres in 2019 and decreased to 0.31 acres in 2020. Comparatively, the crop area under maize and soybean are relatively higher than the area under groundnut and rice cultivation. Except for soybean, all the crops witnessed a decline in the area under cultivation in 2020.

### 5.2 Probit estimates of factors influencing crop land allocation decisions

Table 4 reports the probit estimation of the factors influencing the crop (soybean) land allocation decisions by farmers due to the COVD-19 pandemic. The dependent variable (dummy) measures if farmers were willing to change crop land allocation decision due to the COVD-19 pandemic. The marginal effects are reported while the clustered standard errors are in parentheses. Model 1 is the base model that did not account for political and COVID-19 controls. Model 2 accounted for political controls while model 3 accounted for both political and COVID-19 controls. There is model improvement across the three models based on the Pseudo R-square value. The Pseudo R-square value increased from 0.145 to 0.320 after controlling for both political and COVID-19 variables. The probability of changing cropland allocation decisions is significantly influenced by age, economic active members within the household, distance to input shop, access to research institute, years of farming experience, access to input shop, relationship with a political figure, perception of the negative effect of COVID-19 on agriculture, perception of COVID-19 effect on crop choice, and current cropping due to the COVID-19 pandemic.

**Table 4:** Factors influencing soybean land allocation decision – probit estimates.

Variables	Marginal	Marginal	Marginal
	effect	effect	effect
	(Model 1)	(Model 2)	(Model 3)
Age	-0.007**	-0.007**	-0.005**
	(0.003)	(0.003)	(0.003)
Sex (1=Male)	0.044	0.000	0.044
	(0.086)	(0.085)	(0.068)
Education years	0.008	0.008	0.005
	(0.005)	(0.005)	(0.004)
Males between 15-35 years	0.006	0.001	0.001
	(0.011)	(0.012)	(0.012)
Males between 36-60 years	-0.025	-0.006	-0.004
	(0.028)	(0.029)	(0.025)
Adult economic active members	0.014	0.012	0.014*
	(0.010)	(0.008)	(0.008)
Dependents	-0.003	-0.002	-0.001
	(0.003)	(0.003)	(0.003)
Member of FBO	-0.065*	-0.087**	-0.028
	(0.034)	(0.034)	(0.030)
Distance to input shop	0.009***	0.008***	0.007***
	(0.002)	(0.002)	(0.002)
Access to research institute	0.215***	0.223***	0.193***
	(0.077)	(0.074)	(0.073)
Years of farming	0.009***	0.009***	0.005**
	(0.003)	(0.003)	(0.002)
Access to input shop	0.128**	0.127**	0.101*
	(0.054)	(0.056)	(0.056)
Access to credit facility	-0.108	-0.121**	-0.088
	(0.068)	(0.061)	(0.059)
Access to grain market	-0.009	-0.036	-0.059
	(0.080)	(0.072)	(0.049)
Access to extension services	0.000	0.003	-0.006
	(0.045)	(0.039)	(0.044)
Farm insurance	0.084	0.180	0.222
	(0.144)	(0.160)	(0.155)
Distance to output markets	-0.006**	-0.006*	-0.004
	(0.003)	(0.003)	(0.002)
*Political controls*			
Related to political figure		0.189***	0.143**
		(0.067)	(0.063)
Affiliated to political party		0.074	0.039
		(0.068)	(0.068)
*COVID-19 controls*			
Education on COVID-19			-0.028
			(0.042)
Negative COVID-19 effect on agriculture			0.076*
			(0.043)
COVID-19 influence crop choice			0.152*
			(0.086)
Current cropping due to COVID-19			0.526***
			(0.089)
Pseudo R2	0.145	0.198	0.320
Observations	309	309	309

*Notes*: Standard errors, presented in parentheses, are adjusted for 18 clusters in community.

*** *p* < 0.01,

** *p* < 0.05 and

* *p* < 0.1.

The probability of changing cropland (soybean) allocation decision is 5% lower among older farmers relative to the young farmers. The result suggests that younger farmers are more likely to change their land allocation decisions due to COVID-19. Younger farmers are more likely to take risk relative to older farmers [38] thus are more likely to allocate more land to crops with relatively higher anticipated price increase due to the COVID-19 pandemic. The result suggests that a unit increase in the number of adults economically active household members is likely to increase the probability of making land allocation decisions to soybean by 1.4%. Households with more economic active members are more likely to contribute financially towards more profitable crops, thus the higher the probability of allocating more land to profitable crops. Distance to input shop is positively associated with land allocation decisions. A unit increase in the distance from homestead to input shop increases the probability of allocating more land to soybean by 7%. Long-distance is associated with high transaction costs. A relatively high transaction cost will increase input cost or may prevent farmers from using modern inputs which may affect their production and land allocation decisions. Framers who travel a longer distance to access specific crop inputs are more likely to allocate less land to soybean irrespective of the profitability of the crop. The high cost of transportation may increase the overall cost of production thus reducing the area under cultivation given that households are income constrained.

Access to research institutions is positively associated with land allocation decisions. Farmers who have access to research institutions are 19% more likely to make a land allocation decision relative to farmers who have no access to a research institution. Research institutions serve as a source of agricultural information and capacity building for farmers. Most of the agricultural research institutions support farmers with new technologies that are disseminated by agricultural extension agents. Farmers with adequate and critical information can make more accurate land allocation decisions with minimal losses. Several studies [39,40] have highlighted the importance of critical information in enhancing farm production decisions.

With respect to the political controls, we observed a positive association between farmer connection with a political figure and soybean land allocation decision. Farmers connected to a political figure in their community are 14% more likely to make cropland allocation decisions relative to those who have no relationship with a political figure. Farmers who are related to politicians are more likely to benefit from government projects that provide agricultural input support (e.g., fertilizer and seed) to smallholder farmers. The type of input support is likely to influence cropland allocation decisions. [33], [34], and Seck (2017) have shown that access to a member of parliament or a political leader has an implication on an individual farming decision.

With respect to the COVID-19 controls, the result shows that perception of the negative effect of COVID-19 on agriculture is positively associated with cropland allocation decisions. Some of the negative effects include lack of support from NGOs, high input price and unavailability of some agricultural inputs, unavailability of labor, lack of remittances, limited access to output markets, and low output price. Farmers who perceive that COVID-19 pandemic will affect agricultural production negatively are 8% more likely to make cropland allocation decisions to soybean relative to farmers with different perceptions of COVID-19 on agricultural production. A negative agricultural shock is likely to influence the type of farm investment and the allocation of resources among different crop portfolios. The result confirms the findings of [35] who found that smallholders exposed to negative shocks are less likely to invest in soil fertility and land management practices since the initial investments are high and the benefits can usually only be derived in the future. According to [36], agricultural shocks influence farmers to make risk-averse decisions that have the potential of influencing farm-level performance. Similarly, farmers are more likely to invest in short term improved technologies with lower risk relative to long term investment with higher risk [37].

Farmers who perceive COVID-19 to influence crop choice are 15% more likely to make cropland allocation decision to soybean compared to those who do not perceive COVID-19 to have any influence on crop choice. The data shows that about 47% of the farmers are shifting resources towards soybean production given that prices of soybean are increasing more than the prices of cereals and other legumes like groundnut due to the COVID-19 pandemic in the urban markets. The current cropping practices due to COVID-19 have a significant and positive association with crop land allocation decision. The probability of making cropland allocation decision is 53% higher among farmers whose current crop practices is as a result of the COVID-19 relative to farmers who use different cropping practices. For example, farmers whose current cropping practices (such as intercropping, monocropping or mixed cropping) are as a result of the COVID-19 pandemic are more likely to make cropland allocation decision among the different crops in order to hedge against crop losses.

### 5.3 Factors determining crop land use

Table 5 reports the results of the SUR. The first four columns show the results of the factors influencing land allocated to maize, soybean, groundnut, and rice while the last two columns show the results of the factors influencing cereal and legume land allocation decisions. All the variables presented in Table 5 significantly influenced land allocation decisions except age and sex. The R-squared value indicates that between 15% and 53% of the variations in the land allocation decision is explained by the independent variables (socio-economic, production, political, external shocks, and institutional factors).

**Table 5:** Determinants of crop land use choices (SUR model results).

Variables	Maize	Soybean	Groundnut	Rice	Cereals	Legumes
Age	-0.014	0.015	0.004	-0.005	-0.020	0.019
	(0.017)	(0.012)	(0.010)	(0.008)	(0.019)	(0.016)
Sex (1=male)	0.187	0.337	-0.169	-0.008	0.179	0.169
	(0.471)	(0.319)	(0.283)	(0.213)	(0.518)	(0.430)
Years of education	-0.047	0.059**	0.016	0.010	-0.037	0.076**
	(0.041)	(0.028)	(0.024)	(0.018)	(0.045)	(0.037)
Member of FBO	0.580	0.319	0.914***	0.295*	0.875**	1.233***
	(0.364)	(0.247)	(0.219)	(0.165)	(0.400)	(0.332)
Years of farming	0.041**	-0.011	-0.006	-0.005	0.037	-0.017
	(0.021)	(0.014)	(0.012)	(0.009)	(0.023)	(0.019)
Access to input shop	-0.500	-0.276	-0.141	-0.490**	-0.990**	-0.417
	(0.438)	(0.297)	(0.264)	(0.198)	(0.482)	(0.400)
Distance to input shop	-0.024*	-0.006	-0.010	-0.007	-0.031**	-0.016
	(0.013)	(0.009)	(0.008)	(0.006)	(0.015)	(0.012)
Access to credit	0.395	-0.016	-0.072	-0.010	0.385	-0.088
	(0.358)	(0.242)	(0.215)	(0.162)	(0.393)	(0.326)
Access to grain market	-0.628	0.561*	0.222	0.282	-0.346	0.783*
	(0.485)	(0.329)	(0.292)	(0.220)	(0.534)	(0.443)
Research institution access	-0.444	-0.368	-0.689**	0.079	-0.365	-1.057**
	(0.539)	(0.365)	(0.324)	(0.244)	(0.592)	(0.491)
Farm Insured	3.902***	0.384	0.371	0.077	3.978***	0.755
	(1.070)	(0.725)	(0.643)	(0.484)	(1.177)	(0.976)
Distance to market	0.069***	0.005	0.048***	0.010	0.079***	0.053***
	(0.019)	(0.013)	(0.011)	(0.009)	(0.021)	(0.017)
Number of extension visits	-0.080	0.163	0.103	0.015	-0.065	0.266*
	(0.158)	(0.107)	(0.095)	(0.071)	(0.174)	(0.144)
Soy planting labour	0.092***	0.290***	0.022	-0.009	0.083**	0.312***
	(0.033)	(0.023)	(0.020)	(0.015)	(0.037)	(0.030)
Soy harvesting labour	0.007	-0.075**	0.025	-0.020	-0.013	-0.049
	(0.053)	(0.036)	(0.032)	(0.024)	(0.058)	(0.048)
Maize planting labour	-0.006	-0.062*	-0.029	-0.043*	-0.049	-0.090**
	(0.050)	(0.034)	(0.030)	(0.023)	(0.055)	(0.046)
Maize harvesting labour	0.037	-0.024	0.042	0.045*	0.081	0.019
	(0.056)	(0.038)	(0.033)	(0.025)	(0.061)	(0.051)
Soybean price	-1.964***	-0.603	0.211	-0.228	-2.192***	-0.391
	(0.595)	(0.403)	(0.358)	(0.269)	(0.654)	(0.543)
Maize weeding labour	0.245***	0.045	-0.017	-0.039	0.206**	0.028
	(0.090)	(0.061)	(0.054)	(0.041)	(0.099)	(0.082)
Soy weeding labour	-0.083	-0.137**	0.082	-0.007	-0.090	-0.055
	(0.087)	(0.059)	(0.052)	(0.039)	(0.095)	(0.079)
Inorganic fertilizer (maize)	1.022***	0.149	0.163	0.308*	1.330***	0.312
	(0.356)	(0.241)	(0.214)	(0.161)	(0.391)	(0.325)
Inorganic fertilizer (soybean)	0.218	-0.119	-0.239	0.415**	0.634	-0.357
	(0.425)	(0.288)	(0.255)	(0.192)	(0.467)	(0.387)
Related to political figure	-0.185	-0.247	-0.302	0.126	-0.059	-0.549*
	(0.344)	(0.233)	(0.207)	(0.156)	(0.378)	(0.314)
Affiliated to political party	1.044***	0.183	0.307	0.136	1.180***	0.489
	(0.387)	(0.262)	(0.232)	(0.175)	(0.425)	(0.353)
Education on COVID-19	0.670*	0.286	-0.112	0.094	0.764**	0.175
	(0.347)	(0.235)	(0.209)	(0.157)	(0.382)	(0.317)
1.DumCovAgric	-0.179	0.445*	-0.115	0.315**	0.136	0.329
	(0.337)	(0.228)	(0.203)	(0.152)	(0.371)	(0.307)
Observations (R-squared)	309 (0.38)	309 (0.53)	309 (0.23)	309 (0.15)	309 (0.38)	309 (0.49)

Note: Standard errors in parentheses.

*** *p* < 0.01,

** *p* < 0.05 and

* *p* < 0.1.

With respect to the socioeconomic factors, years of education is associated with a positive effect on land allocated to soybean and legumes in general. This indicates that educated farmers are more likely to allocate more of the cropland to legume production. A unit increase in the years of formal education increases the land allocated to soybean and legume by 0.06 acres and 0.08 acres, respectively. Education empowers individuals to make an informed decision about food security and income. The result contradicts the findings of [41] who found that primary education attainment increases the land allocated to cereals. On the contrary, [42] showed that educated household heads allocate less land to maize and more to other crops (groundnut, beans, and all other annual crops) than households where the head has no education. Our result suggests that educated farmers are more likely to make decisions that will favor commercial crops relative to food crops.

Member of farmer association is significant and positively associated with both cereal and legume land allocation. Compared to non-members of FBO, farmers that belong to FBOs are more likely to increase the land allocated to cereals and legumes by 0.88 acres and 1.23 acres, respectively. Group membership guarantees access to agricultural inputs, credit, knowledge sharing, and support for members. Most farmer groups are based on specific crops that may influence the land allocation decisions. Our result is consistent with the findings of [43] and [6] who found a positive relationship between group membership and land allocated to legumes and cereals, respectively.

For institutional factors, farmers who have access to input shop decreases the land allocated to cereals by 0.99 acres compared to farmers who do not have access to the input shop. Similarly, distance to input shop which captures transaction cost reduces the area of land allocated to cereals by 0.03 acres. Costs related to high transaction costs associated with distance and high cost of inputs may discourage farmers from expanding their farms. Despite the importance of credit in agricultural production decisions, our results show no significant effect of credit on cropland allocated to both legumes and cereals. However, some studies have shown that credit access increase area allocated to other annual crops in Thailand [6] and cash crops in Benin [8].

Compared to farmers who do not have access to grain markets, farmers who have access to grain market increases the land allocated to legumes by 0.78 acres. A readily available market reduces farmers’ high risks of theft, postharvest losses, and high cost of storage. Contrary to our expectations, farmers with access to research institutions reduce their area under legume cultivation by 1.06 acres. Research institutions have been at the forefront of agricultural technology development. Farmers may have access to research institutions but may not have access to the agricultural technologies related to legumes thus are more likely to reduce their land allocation. In recent times, there has been strong advocacy for sustainable land intensification for effective monitoring and management of small farms [38]. Farmers may be responding to this call by reducing the area under crop cultivation. [21] found that crop complexity or newness reduces the crop area allocated to soybean cultivation in Ghana.

Farmers who have formally subscribed to an insurance policy against the risk of crop failure are more likely to increase the area under cereal production by 3.98 acres. Farm insurance serves as a risk-reducing mechanism which allows farmers to expand given that they do not bear the entire risks of crop failure, theft, and low output price. In order to estimate the effect of the price at different markets, we use farmer geo-codes to estimate the distance to the nearest market where they either buy or sell food commodities for household consumption. Distance to output markets reflects different output prices faced by the farmers. The area allocated to cereal and legume production increases by 0.08 acres and 0.05 acres, respectively for a unit increase in the distance to output markets. The results suggest that farmers are responding appropriately by allocating more land to cereal production relative to legume production. This could be a case where household objective is to enhance food security relative to investment in cash crop. The number of extension visits increases the area allocated to soybean by 0.27 acres. Contrary to our expectation, [8] found a reverse relationship between extension access and area allocated to cereal production. The measurement of extension access may be a contributing factor given that they use a dummy variable to capture access while we use the number of extension visits.

With respect to production factors, the number of laborers used for sowing soybean seeds increases the area under cereal and legume production by 0.08 acres and 0.31 acres, respectively. The result confirms the theory of joint production where an increase in the labor input of soybean increases the labour input of maize. The number of laborers used for sowing maize seeds decreases the area under legume production by 0.09 acres with no significant effect on the maize production area. Consistent with the a priori expectation, an increase in the price of soybean decreases the area under cereal production by 2.19 acres. Farmers are more likely to increase the area under legume production when they expect the price of legumes to increase relative to the price of cereals. The amount of labor available for weeding the maize plot and users of inorganic fertilizer on maize plot increases the area under cereal production by 0.21 acres and 1.33 acres, respectively. The result is consistent with the findings of [8] who found a positive association between fertilizer use and cereal land allocation.

Political factors such as the relation with a political figure and affiliation to a political party significantly influenced land allocation decisions. [33,34], and Seck (2017) found that access to a Member of Parliament or political leaders has an implication on an individual farming decision. Farmers who are related to a political figure are more likely to reduce the land allocated to legume by 0.55 acres relative to farmers who are not related to a political figure. Political affiliation increases land allocation to cereals by 1.18 acres. The result is not surprising given that political affiliation guarantees access to developmental projects that provide input support to smallholder farmers. The current policy on “planting for food and job” provides subsidized inputs to farmers. Most of the government development interventions target maize farmers; thus, farmers who are affiliated to the political party in power have a higher chance of getting such input supports. Farmers who have the assurance of getting agricultural input support will be more willing to allocate more land to cereal production relative to other crops. Given that most of the projects target maize production, farmers who are related to political figures may shift land resources towards maize production at the expense of legumes thus the negative association with legumes.

For the negative external shocks, farmers who are educated about the COVID-19 pandemic are more likely to increase the land allocated to cereals by 0.76 acres relative to framers who are uneducated about the COVID-19 pandemic. Similarly, farmers who consider COVID-19 pandemic to have a negative effect on agricultural production is likely to increase the area under soybean and rice by 0.45 acres and 0.32 acres, respectively. A farmer who is more informed about the impact of the COVID-19 pandemic on agricultural production is more likely to make informed decisions with respect to input and output choices. The measures put in place to contain the spread of the virus disrupted the supply of agro-food products to markets and consumers leading to a shift in the demand for some food relative to the others [44]. The markets for cereals are likely to be less affected compared to the perishable markets due to the disruptions in the transportation system as well as agricultural labor markets. In view of this, farmers may respond by allocating more land to cereals relative to other crops. As a short-term mechanism, farmers may be more focused on meeting their household food demand while maximizing their income in the long term by allocating more land to legume production. Reduction in the number of buyers who trade with smallholder farmers due to the restriction imposed by the government of Ghana may be accounting for the disruptions in the land allocation decision between cereals and legumes. Generally, the findings of the study confirm the negative effect of the COVID-19 pandemic on crop choices among rural households in Ghana.

## 6 Conclusion

The novel COVID-19 pandemic is a global health issue that has resulted in huge economic shocks leading to short-term disruptions in the agricultural food systems with possible dire long-term effects. Farmers are more likely to benefit from the external shock of COVID-19 by shifting land resources between staples and cash crops depending on the objective of the household. This study extends the literature on land resource allocation by accounting for the effects of the COVID-19 pandemic on land allocation decisions among soybean farmers in the Northern Region of Ghana. The study employs both the probit and seemingly unrelated regression model to identify the factors influencing land allocation decisions and the size of cropland allocated to legumes and cereals. The results revealed that cropland allocation decisions are significantly influenced by age, economic active members within the household, distance to input shop, access to research institute, years of farming experience, access to input shop, relationship with a political figure, perceived negative effects of COVID-19 on agriculture, perceived COVID-19 effects on crop choice, and current cropping practices due to the COVID-19 pandemic.

The size of cropland allocated to legumes and cereals are determined by socio-economic, production, political, external shocks, and institutional factors. More specifically, regarding crop land use, the significant determining factors are years of education, member of farmer association, access to input shop, access to a research institution, farm insurance, distance to output markets, number of extension visits, labor for planting soybean and maize, price of soybean, access to inorganic fertilizer, relation with a political figure and affiliation to a political party, education on COVID-19 pandemic, and perceived negative effects of COVID-19 on agricultural production.

The findings of our study highlight several important implications that are relevant to smallholder farmers, development practitioners, and policymakers. Differences in commodity prices at different output markets correlate highly with cropland allocation decisions. Improvement in transportation infrastructure and feeder roads connecting farms to major roads are necessary for reducing the transaction cost incurred by smallholder farmers. The transactional cost has implications on crop choice decisions made by farm households and consequently, welfare outcomes. Access to inorganic fertilizer increases the area under cereal cultivation. This finding suggests that the government of Ghana's flagship program on “planting for food and jobs” that provides a 50% subsidy on the cost of seeds and fertilizers to smallholder farmers must be sustained. Critical to this study is the positive effects of COVID-19 education and the perceived negative effects of COVID-19 on crop land-use decisions. Development practitioners must intensify farmer education on the disruptive effects of the COVID-19 pandemic on agricultural production and the strategies that can be employed to mitigate the negative impact. Understanding local farmer perceptions of the disruptive effects of the COVID-19 pandemic on agriculture will be critical to guide future adaption strategies, campaigns, and possible mitigation efforts. The provision of accurate and timely information on the disruptive effects of the COVID-19 pandemic would enable farmers to determine the “best” possible cropland allocation decision.

## Authors’ contribution

All persons who meet authorship criteria are listed as authors, and all authors certify that they have participated sufficiently in the work to take public responsibility for the content, including participation in the concept, design, analysis, writing, or revision of the manuscript. Furthermore, each author certifies that this material or similar material has not been and will not be submitted to or published in any other publication before its appearance in the *Sustainable Futures*.

## Declaration of Competing Interest

The authors declare that they have no known competing financial interests or personal relationships that could have appeared to influence the work reported in this paper.

## Footnotes

1 https://statsghana.gov.gh/gssmain/fileUpload/Price%20Indices/Bulletin_%20CPI%20May%202020.pdf.

2 The utility that farmers obtain from different crops is likely to, for example, differ by age and educational status and therefore influence land allocation decisions.

## References

[1] Afridi F., Dhillon A., Roy S. (2020). How has Covid-19 Crisis Affected the Urban Poor? Findings from a Phone Survey. Ideas for India.

[2] Laborde D., Martin W., Swinnen J., Vos R. (2020). COVID-19 risks to global food security. Science.

[3] Kom Z., Nethengwe N.S., Mpandeli N.S., Chikoore H. (2020). Determinants of small-scale farmers’ choice and adaptive strategies in response to climatic shocks in Vhembe District, South Africa. GeoJournal.

[4] Mitter H., Heumesser C., Schmid E. (2015). Spatial modeling of robust crop production portfolios to assess agricultural vulnerability and adaptation to climate change. Land use policy.

[5] Birthal P.S., Roy D., Negi D.S. (2015). Assessing the impact of crop diversification on farm poverty in India. World Development.

[6] Arouri M., Nguyen C., Youssef A.B. (2015). Natural disasters, household welfare, and resilience: evidence from rural Vietnam. World development.

[7] Seck A. (2017). Fertiliser subsidy and agricultural productivity in Senegal. The World Economy.

[8] Nguyen T.T., Nguyen L.D., Lippe R.S., Grote U. (2017). Determinants of farmers’ land use decision-making: Comparative evidence from Thailand and Vietnam. World Development.

[9] Porgo M., Kuwornu J.K., Zahonogo P., Jatoe J.B.D., Egyir I.S. (2018). Credit constraints and cropland allocation decisions in rural Burkina Faso. Land Use Policy.

[10] Kokoye S.E.H., Tovignan S.D., Yabi J.A., Yegbemey R.N. (2013). Econometric modeling of farm household land allocation in the municipality of Banikoara in Northern Benin. Land use policy.

[11] Msongaleli B., Tumbo S., Rwehumbiza F., Kihupi N. (2015). Determinants of farm-level decisions regarding cereal crops and varieties in semi-arid central Tanzania. African Journal of Agricultural Research.

[12] Elbehri A., Kaminski J., Koroma S., Iafrate M., Benali M., Elbehri A. (2013). Rebuilding West Africa's Food Potential.

[13] Finnis E. (2006). Why grow cash crops? Subsistence farming and crop commercialization in the Kolli Hills, South India. American Anthropologist.

[14] Messina M.J. (1999). Legumes and soybeans: overview of their nutritional profiles and health effects. The American Journal of Clinical Nutrition.

[15] Muoni T., Barnes A.P., Öborn I., Watson C.A., Bergkvist G., Shiluli M., Duncan A.J. (2019). Farmer perceptions of legumes and their functions in smallholder farming systems in east Africa. International Journal of Agricultural Sustainability.

[16] Ojiewo C., Keatinge D.J.D.H., Hughes J., Tenkouano A., Nair R., Varshney R., Siambi M., Monyo E., Ganga-Rao N.V.P.R., Silim S. (2015). The role of vegetables and legumes in assuring food, nutrition, and income security for vulnerable groups in sub-Saharan Africa. World Medical and Health Policy.

[17] Hassen A., Talore D.G., Tesfamariam E.H., Friend M.A., Mpanza T.D.E. (2017). Potential use of forage-legume intercropping technologies to adapt to climate-change impacts on mixed crop-livestock systems in Africa: A review. Regional Environmental Change.

[18] FAO (2016).

[19] Thierfelder C., Cheesman S., Rusinamhodzi L. (2012). A comparative analysis of conservation agriculture systems: Benefits and challenges of rotations and intercropping in Zimbabwe. Field Crops Research.

[20] Bationo A., Waswa B., Okeyo J.M., Maina F., Kihara J., Mokwunye U. (2011).

[21] Odendo M., Bationo A., Kimani S., Bationo A., Waswa B., Okeyo J.M., Maina F., Kihara J., Mokwunye U. (2011). Fighting poverty in Sub-Saharan Africa: The multiple roles of legumes in integrated soil fertility management.

[22] Chivenge P., Mabhaudhi T., Modi A.T., Mafongoya P. (2015). The potential role of neglected and underutilized crop species as future crops under water scare conditions in sub-Saharan Africa. International Journal of Environmental Research and Public Health.

[23] Tamimie C.A., Goldsmith P.D. (2019). Determinants of soybean adoption and performance in Northern Ghana. African Journal of Agricultural and Resource Economics.

[24] Waldman K.B., Ortega D.L., Richardson R.B., Clay D.C., Snapp S. (2016). Preferences for legume attributes in maizelegume cropping systems in Malawi. Food Security.

[25] Shelton H.M., Franzel S., Peters M. (2015). Adoption of tropical legume technology around the world: Analysis of success. Tropical Grasslands.

[26] Bezner Kerr R.B., Snapp S., Chirwa M., Shumba L., Msachi R. (2007). Participatory research on legume diversification with Malawian smallholder farers for improved nutrition and soil fertility. Experimental Agriculture.

[27] Mpepereki S., Javaheri F., Davis P., Giller K.E. (2000). Soyabeans and sustainable agriculture: promiscuous soyabeans in southern Africa. Field crops research.

[28] Olwande J., Smale M., Mathenge M.K., Place F., Mithöfer D. (2015). Agricultural marketing by smallholders in Kenya: A comparison of maize, kale and dairy. Food Policy.

[29] Jayne T.S., Chamberlin J., Headey D.D. (2014). Land pressures, the evolution of farming systems, and development strategies in Africa: A synthesis. Food policy.

[30] Kruseman G., Ruben R., Kuyvenhoven A., Hengsdijk H., Van Keulen H. (1996). Analytical framework for disentangling the concept of sustainable land use. Agricultural Systems.

[31] Quan J. (1998). Land tenure and sustainable rural livelihoods. Sustainable Rural Livelihoods-What Contribution We Make.

[32] Manda J., Alene A.D., Gardebroek C., Kassie M., Tembo G. (2016). Adoption and impacts of sustainable agricultural practices on maize yields and incomes: evidence from Rural Zambia. Journal of Agricultural Economics.

[33] Van Campenhout Bjorn, Walukano Wilberforce, Nattembo Fiona, Nazziwa-Nviiri Lydia, Blom Jaap (2017). http://ebrary.ifpri.org/cdm/singleitem/collection/p15738coll2/id/131516.

[34] Jack B.Kelsey (2013). Constraints on the adoption of agricultural technologies in developing countries.” Literature review, Agricultural Technology Adoption Initiative. J-PAL (MIT) and CEGA (UC Berkeley.

[35] Ragasa C., Mazunda J. (2018). The impact of agricultural extension services in the context of a heavily subsidized input system: The case of Malawi. World development.

[36] Ricker-Gilbert J., Jayne T.S., Chirwa E. (2011). Subsidies and crowding out: A double-hurdle model of fertilizer demand in Malawi. American journal of agricultural economics.

[37] Shikuku K.M., Winowiecki L., Twyman J., Eitzinger A., Perez J.G., Mwongera C., Läderach P. (2017). Smallholder farmers’ attitudes and determinants of adaptation to climate risks in East Africa. Climate Risk Management.

[38] Adjimoti G.O. (2018). Analysis of cropland allocation in rural areas Benin: A fractional multinomial approach. Cogent Food & Agriculture.

[39] Chibwana C., Fisher M., Shively G. (2012). Cropland allocation effects of agricultural input subsidies in Malawi. World Development.

[40] Struik P.C., Kuyper T.W. (2017). Sustainable intensification in agriculture: the richer shade of green. A review. Agronomy for Sustainable Development.

[41] OECD Policy Responses to Coronavirus (COVID-19). (2020). COVID-19 and the food and agriculture sector: Issues and policy responses. https://read.oecd-ilibrary.org/view/?ref=130_130816-9uut45lj4q&title=Covid-19-and-the-food-and-agriculture-sector-Issues-and-policy-responses.

[42] Waldman K.B., Blekking J.P., Attari S.Z., Evans T.P. (2017). Maize seed choice and perceptions of climate variability among smallholder farmers. Global Environmental Change.

[43] Dedeurwaerdere T., Hannachi M. (2019). Socio-economic drivers of coexistence of landraces and modern crop varieties in agro-biodiversity rich Yunnan rice fields. Ecological economics.

[44] Eldukhery I., Elamin N.H., Kherallah M., Abur A.T. (2010).

